# Is There a Place for Family-centered Cesarean Delivery during Placenta Accreta Spectrum Treatment?

**DOI:** 10.1055/s-0042-1751060

**Published:** 2022-09-06

**Authors:** Albaro José Nieto-Calvache, Alejandra Hidalgo, Juliana Maya, Beatriz Sánchez, Luisa Fernanda Blanco, Stiven Ernesto Sinisterra-Díaz, Juan Pablo Benavides-Calvache, Iván Padilla, Ivonne Aldana, Martha Jaramillo, Ana Maria Gómez, Angela María Olarte Castillo, Adriana Messa Bryon

**Affiliations:** 1 Clínica de Espectro de Acretismo Placentario, Fundación Valle del Lili, Cali, Colombia; 2 Universidad Icesi, Programa de Medicina, Cali, Colombia; 3 Departamento de Anestesiología, Fundación Valle del Lili, Cali, Colombia; 4 Centro de Investigaciones Clínicas, Fundación Valle del Lili, Cali, Colombia; 5 Unidad de Cuidad Intensivo Neonatal, Fundación Valle del Lili, Cali, Colombia; 6 Unidad de Cuidado Intensivo Pediátrico, Fundación Valle del Lili, Cali, Colombia

**Keywords:** placenta accreta, humanization, birthing centers, patient-centered care, placenta acreta, humanização, centros de parto, cuidado centrado no paciente

## Abstract

**Objective** Placenta accreta spectrum (PAS) is a cause of massive obstetric hemorrhage and maternal mortality. The application of family-centered delivery techniques (FCDTs) during surgery to treat this disease is infrequent. We evaluate the implementation of FCDTs during PAS surgeries.

**Methods** This was a prospective, descriptive study that included PAS patients undergoing surgical management over a 12-month period. The patients were divided according to whether FCDTs were applied (group 1) or not (group 2), and the clinical outcomes were measured. In addition, hospital anesthesiologists were surveyed to evaluate their opinions regarding the implementation of FCDTs during the surgical management of PAS.

**Results** Thirteen patients with PAS were included. The implementation of FCDTs during birth was possible in 53.8% of the patients. The presence of a companion during surgery and skin-to-skin contact did not hinder interdisciplinary management in any case.

**Conclusion** Implementation of FCDTs during PAS care is possible in selected patients at centers with experience in managing this disease.

## Resumo

**Objetivo** O espectro da placenta acreta (do inglês *placenta accreta spectrum* - PAS) é causa de hemorragia obstétrica maciça e mortalidade materna. A aplicação de técnicas de parto centrado na família (do inglês *family-centered delivery techniques* - FCDTs) durante a cirurgia para tratar esta doença é pouco frequente. Avaliamos a implementação das FCDTs durante as cirurgias do PAS.

**Métodos** Estudo prospectivo e descritivo que incluiu pacientes com PAS submetidos a tratamento cirúrgico durante um período de 12 meses. Os pacientes foram divididos de acordo com a aplicação de FCDTs (grupo 1) ou não (grupo 2), e os resultados clínicos foram medidos. Além disso, anestesiologistas hospitalares foram entrevistados para avaliar suas opiniões sobre a implementação das FCDTs durante o manejo cirúrgico do PAS.

**Resultados** Foram incluídos 13 pacientes com PAS. A implementação de FCDTs durante o parto foi possível em 53,8% das pacientes. A presença do acompanhante durante a cirurgia e o contato pele a pele não prejudicou o manejo interdisciplinar em nenhum caso.

**Conclusão** A implementação de FCDTs durante o atendimento do PAS é possível em pacientes selecionados em centros com experiência no manejo dessa doença.

## Introduction

Placenta accreta spectrum (PAS) is a cause of maternal morbidity. 1 Its management may require highly complex interventions, including prolonged surgeries with extensive dissection and massive transfusions. 2 Those conditions make general anesthesia the most widely used anesthetic method for PAS surgery, especially in low- and middle-income countries. 3 4 Although the benefits of family-centered delivery techniques (FCDTs), such as having a companion during the surgical procedure or allowing skin-to-skin contact between neonates and the mother, have been demonstrated when part of a routine cesarean section, 5 6 7 8 their use in PAS surgery is infrequent.

In patients affected by PAS, being aware of the possibility of suffering from serious complications (even death) and being subjected to highly complex interventions have been found to be related to posttraumatic stress disorder 9 and to the request for psychological support. 10

We evaluate the implementation of FCDTs during PAS surgeries, as well as the opinions of a group of anesthesiologists about this activity.

## Methods

A prospective descriptive study was conducted between August 2018 and March 2020. We included all patients undergoing PAS surgical management on a scheduled basis, after a prenatal diagnosis was established and interdisciplinary surgery planning at the Fundación Valle de Lili (FVL), Cali, Colombia. Cases with an intraoperative diagnosis of PAS were excluded.

Details of the anesthetic and surgical protocols are described elsewhere. 11 12 Details about the FCDTs applied (having a companion during the surgery and skin-to-skin contact between the newborn and the mother) are described in Chart 1 .

**Chart 1:** Details of the presence of a companion in the operating room and skin-to-skin contact

In August 2018, the decision was made to favor FCDT among patients with PAS at FVL. Two interventions were proposed to the anesthesiologists and neonatologists: the presence of a companion during the surgery and skin-to-skin contact between the newborn and her mother. The final decision to allow these two strategies in a specific case rested directly with the treating anesthesiologist. The presence of a companion in the operating room was allowed when the surgery was performed under scheduled conditions, in the absence of active vaginal bleeding, and with normal vital signs at the beginning of the surgery. The patient and her companion were educated on the interventions to be performed during the surgery and informed that if the patient's clinical condition deteriorated, it would be necessary for the companion to leave the operating room. In all cases managed with neuraxial anesthesia, at the time of fetal extraction, the newborn was presented to her mother, and skin-to-skin contact was favored when the newborn's condition was stable. Hence, the baby was delivered to the neonatologist who performed the neonatal adaptation on the mother's breast, involving the companion when possible. The companion was removed from the operating room when the newborn was transferred to the recovery room or the neonatal unit. The cases attended in the study period in which these strategies were applied were recorded. The time between birth and breastfeeding, the time between birth and visual or tactile contact between the newborn and mother, and whether the anesthesiologist perceived that the surgical or anesthetic procedure was hampered by the presence of the companion were evaluated.

Abbreviations: FCDT, family-centered delivery techniques; FVL, Fundación Valle de Lili; PAS, placenta accreta spectrum.

In July 2019, a survey was conducted among all FVL anesthesiologists seeking their opinion about the application of FCDTs during the surgical management of PAS.

We divided the patients into those who had skin-to-skin contact with their neonates and/or the presence of a relative during the surgery (group 1) and those who did not (group 2). The clinical results were compared between the two groups, as were the mother-neonate interaction and the anesthesiologist's perception of the impact of FCDTs on the anesthetic procedure.

Continuous variables were expressed as central tendency measurements (median) and dispersion measurements (interquartile range [IQR]) based on normal distribution criteria. Categorical variables were expressed as absolute and relative frequencies.

The ethics committee of FVL approved the study protocol (protocol no. 1023), and informed consents were obtained from all the study participants.

## Results

During the study period, 16 patients with suspected PAS were treated. Three of them had an intraoperative diagnosis and were excluded, and the remaining 13 patients were included in the analysis. In 7 patients (53.8%), FCDTs were applied (group 1), while the remaining 6 patients underwent the surgery unaccompanied, and skin-to-skin contact was not allowed (group 2). Table 1 presents the opinions of the 28 FVL anesthesiologists about the application of FCDTs during the care of PAS patients

Table 2 shows the clinical outcomes of groups 1 and 2.

**Table 1:** Opinions of anesthesiologists on FCDT during the care of patients with PAS ( *n* = 28)

Variable	n (%)
Are you familiar with FCDT employed during c-section birth?	
● Yes	26 (92.8)
What importance do you give to FCDT during birth? *	5 (4–5) **
Do you apply FCDT during elective c-section?	
● Yes	26 (92. 8)
Do you apply FCDT during emerging c-section?	
● Yes	14 (50)
What is your preferred type of anesthesia during the management of PAS?	
● Neuraxial	27 (96.4)
● General	1 (3.6)
Would you allow the presence of a companion during PAS surgery?	
● Yes	7 (25)
Would you allow skin-to-skin contact during PAS surgery?	
● Yes	10 (35.7)
What are your reasons for not allowing a companion during the PAS surgery?	
• High risk of complications	19 (67.8)
• The presence of a companion hinders the work of health professionals during the management of complications • Caring for the companion can become one more task for an already busy team • During this surgery, information is handled that can be misinterpreted by the companion	2 (7.1) 3 (10.7) 1 (3.6)
Would skin-to-skin contact hinder the proper management of the patient affected by PAS?	
• Yes	5 (17.8)
If you currently do not allow FCDT, in what situation would you allow them in cases of PAS?	
• Under no condition	17 (60.7)
• Only after the severity of the lesion has been determined, and control of the situation has been established	4 (14.3)
• Only if clear criteria are established for FCDT implementation, including time limits for each of the interventions	2 (7.1)
• Personnel in charge of supervising these interventions (skin-to-skin contact and companion in the room) are present	5 (17.9)

Abbreviations: FCDT, family-centered delivery techniques; PAS, placenta accreta spectrum.

* score from 1 to 5, with 1 representing “not important” and 5 representing “very important.”

** medium (interquartile range).

**Table 2:** Clinical characteristics of the patients, according to the strategy used for emotional support to the patient during birth

Variables	Group 1 ( *n* = 7)	Group 2 ( *n* = 6)
Gestational age (weeks) *	36 (34–38)	34.5 (33–37)
Histological confirmation of PAS. N (%) **	6 (85.7)	5 (83.3)
Intraoperative blood loss (milliliters) *	1,048 (459–1,480)	1,578 (1,053–2,055)
RBCU transfusion. N (%)	3 (42.8)	2 (33.3)
Hysterectomy. n (%)	4 (57.1)	3 (50)
Uterine tamponade. N (%)	2 (28.5)	3 (50)
Uterine tourniquet. N (%)	5 (71.4)	5 (83.3)
Uterus compression sutures. N (%)	2 (28.5)	3 (50)
Aortic occlusion. n (%)	2 (28.5)	3 (50)
Uterus S2 involvement. N (%) ***	4 (57.1)	4 (66.6)
Neuraxial anesthesia. N (%)	7 (100)	3 (50)
Duration of surgery (minutes) *	195 (172–220)	178 (135–233)
Neonate weight (grams) *	2,713 (1,846–2,885)	2,564 (2,109–2,968)
APGAR at 10 minutes *	8 (8–8)	9 (8–9)
Hospitalization of the newborn. N (%)	3 (42.8)	3 (50)

Abbreviations: PAS, placenta accreta spectrum; RBCU, red blood cell units.

* median (Interquartile Range).

** accreta, increta, or percreta.

*** involvement of the cervix, parametria, or lower uterine segment.

Table 3 shows the differences in the interaction between mother and neonate between groups 1 and 2.

**Table 3:** Results observed with the strategy used for emotional support of the patient during birth

Variable	Group 1 ( *n* = 7)	Group 2 ( *n* = 6)
Mother-baby skin contact in the operating room. N (%)	7	0
Companion-baby skin contact in the operating room. N (%)	3	0
Presence of a companion during surgery. N (%)	5	0
Time between birth and mother-newborn contact (hours) *	0	6.5 (4–36)
Breastfeeding within the first 12 hours of birth. N (%)	3	3
Time between birth and breastfeeding (hours) *	8 (2–44)	28 (4–144)
Anesthesiologist report of additional difficulty of anesthetic management due to the implementation of FCDT by the management team. N (%)	0	Does not apply

Abbreviation: FCDT: family-centered delivery techniques.

* median (interquartile range);

## Discussion

The presence of a companion during surgery and skin-to-skin contact did not hinder anesthetic management in any case ( Table 3 ). Similar to observations in other high-risk obstetric populations, 13 the implementation of FCDTs has difficult challenges to overcome, such as the burden of responsibility for the health of the mother and fetus, the added challenges posed by the presence of a companion, and the fear of legal conflicts about complications during surgery ( Table 1 ).

Although almost all of the anesthesiologists surveyed ( *n* = 26, 92.8%) stated that they were familiar with FCDTs and applied them during elective cesarean sections ( Table 1 ), only 25% and 35.7% of them reported that they would allow the presence of a companion and skin-to-skin contact during PAS surgery, respectively ( Table 1 ). Furthermore, during the period studied, only 7 of the 13 patients with PAS undergoing surgery benefited from skin-to-skin contact with their neonates. Among those patients, only 5 had a companion present during the surgical procedure.

In some PAS reference centers, the frequency of neuraxial anesthesia can be as high as 95%, 14 which allows the mother to be alert, as she anxiously awaits the resolution of a serious disease, and the birth of her child. Although greater maternal satisfaction with the experience of childbirth has been reported when a companion is allowed during the cesarean section, 15 and although benefits are reported in terms of breastfeeding, neonatal temperature regulation, and neonatal cutaneous bacterial colonization when maternal-neonatal skin contact is made within the first hour of life, 5 8 these observations are often ignored during “complicated” cesarean sections.

The most common reason mentioned by the anesthesiologists surveyed for not allowing a companion in surgery was the high risk of complications in this type of surgery ( *n* = 19, 67.8%). However, none of the 13 patients who were treated on a scheduled basis due to prenatal suspicion of PAS had sudden or uncontrollable massive bleeding.

The management of PAS in developing countries faces difficulties much higher in a priority list than the use of FCDT. 16 However, even in pregnancies with serious conditions, it is worth asking whether there is a place for FCDT, especially given that posttraumatic stress disorder is reported in 40% of patients with PAS, 9 which highlights the importance to improve these women's birthing experience , and considering that the presence of a companion during surgery or skin-to-skin contact was not perceived by the anesthesiologist as hindering the normal performance of the activities necessary for PAS treatment among any patients in group 1.

Our study has limitations; we described a small number of cases from a single center, so that the external validity of our observations depends on the strategies used in other centers. Additionally, selection bias during candidates' selection for FCDTs cannot be ruled out. However, this study provides an initial observation of the viability of FCDTs during surgeries for selected cases of PAS. Finally, although we assume a benefit of applying FCDTs in terms of psychological impact during the surgical management of PAS, our study does not have the capacity to evaluate these types of implications. We only intend to draw attention to the issue, as multicenter studies are necessary to evaluate the receptivity of PAS teams to this type of intervention as well as the possible benefits for patients, their neonates, and their families.

## Conclusion

Applying FCDTs during PAS care is possible in selected patients at centers with experience in managing this disease.
